# Multi-sensor Fusion Road Friction Coefficient Estimation During Steering with Lyapunov Method

**DOI:** 10.3390/s19183816

**Published:** 2019-09-04

**Authors:** Letian Gao, Lu Xiong, Xuefeng Lin, Xin Xia, Wei Liu, Yishi Lu, Zhuoping Yu

**Affiliations:** 1 School of Automotive Studies, Tongji University, Shanghai 201804, China (L.G.) (X.L.) (X.X.) (W.L.) (Y.L.) (Z.Y.); 2 Clean Energy Automotive Engineering Center, Tongji University, Shanghai 201804, China

**Keywords:** road friction coefficient, tire model, nonlinear observer, self-aligning torque, lateral displacement, Lyapunov method

## Abstract

The road friction coefficient is a key parameter for autonomous vehicles and vehicle dynamic control. With the development of autonomous vehicles, increasingly, more environmental perception sensors are being installed on vehicles, which means that more information can be used to estimate the road friction coefficient. In this paper, a nonlinear observer aided by vehicle lateral displacement information for estimating the road friction coefficient is proposed. First, the tire brush model is modified to describe the tire characteristics more precisely in high friction conditions using tire test data. Then, on the basis of vehicle dynamics and a kinematic model, a nonlinear observer is designed, and the self-aligning torque of the wheel, lateral acceleration, and vehicle lateral displacement are used to estimate the road friction coefficient during steering. Finally, slalom tests and DLC (Double Line Change) tests in high friction conditions are conducted to verify the proposed estimation algorithm. Test results showed that the proposed method performs well during steering and the estimated road friction coefficient converges to the reference value rapidly.

## 1. Introduction

Vehicle safety-related state estimation [1,2,3] and control systems [4,5,6] have received much attention in recent decades. Vehicle dynamic control systems, such as the ASR (Anti-slip Regulation System), ESC (Electronic Stability Control), and AEB (Autonomous Emergency Brake), are realized by controlling the driving forces or braking forces so that the forces exerted by the road on the tires can be changed to maintain the stability of the wheels and the vehicle. The road friction coefficient is a key parameter for vehicle dynamic control systems [7,8], because it can reflect the dynamic motion limitations to a certain extent [9]. For human-operated vehicles, drivers can estimate the motion limitation of the vehicle and adapt their driving style using experience to prevent the vehicle from driving into critical conditions. However, with the development of intelligent vehicles, progressively more ADAS (Advanced Driving Assistant System) functions are being implemented by automated systems, which means that driving and braking forces and steering angles need to be calculated and controlled by control units, such as ACC (Adaptive Cruise Control) and LKA (Lane Keep Assistant). Therefore, an accurate road friction coefficient provides the automated system with the current motion limitation of the vehicle. Furthermore, for highly automated vehicles, the road friction coefficient is critical for decision-making, trajectory planning, and trajectory tracking.

Road friction coefficient estimation methods can be divided into two general types: cause-based methods and effect-based methods. The state-of-the-art methods of road friction estimation have been reviewed [10]. The principle of cause-based methods is the direct determination of road surface characteristics by special sensors, such as cameras, laser scanners, optical sensors, and so on. Alonso, J. et al. [11] proposed an asphalt status classification system based on real-time acoustic analysis of the tire-road interaction, but only wet and dry asphalt states were covered. Roychowdhury, S. et al. [12] proposed a two-stage method based on images captured by the front camera. A convolutional neural network model was applied to learn the road characteristics, and then the road states were divided into three types according to a rule-based strategy. Although cause-based methods can accurately characterize road states with special sensors, only a rough road friction coefficient is estimated, and the interval may vary within a very large range. The road friction coefficient describes the interaction effects between the road and tires [13], which means it cannot be estimated accurately by cause-based methods without considering the vehicle and tire characteristics. Effect-based methods estimate the road friction coefficient from the dynamic or kinematic motion responses [14] of the vehicle or wheels due to the tire forces caused by the interaction between the tires and the road. Normally, effect-based methods estimate the road friction coefficient by using models, and the estimation results are more accurate than those of cause-based methods. Effect-based methods fall into two categories: methods based on longitudinal dynamics and methods based on lateral dynamics. In order to obtain the road friction coefficient in all conditions, Ahn, C. et al. [15] divided driving conditions according to the slip ratio, the sideslip angle, and lateral acceleration. Then, different estimation methods were applied to estimate road friction coefficients under different conditions. Using longitudinal dynamics, Castillo Aguilar, J.J. et al. [16] applied a fuzzy logic algorithm to estimate the road friction according to the variation curve of the relationship between the road friction coefficient and the longitudinal slip ratio, and the algorithm was utilized in the hydraulic pressure control system of the EHB (Electric Hydraulic Brake) [17]. Enisz, K. et al. [18] designed an augmented vehicle model with the road friction coefficient and slip ratio, and the vehicle speed, wheel rotation speed, slip ratio, and road friction coefficient were simultaneously estimated by the EKF (Extended Kalman Filter). Taking advantage of the fact that the wheel torque of distributed drive electric vehicles is available and can be controlled precisely, Xia, X. et al. [19] proposed a road friction coefficient estimation algorithm under driving conditions using a nonlinearobserver. The observer performed well with strong longitudinal excitation, and its stability was proved. Moreover, many studies have focused on road friction coefficient estimation methods based on lateral dynamic characteristics. Using the relationship between lateral force and the sideslip angle, Wang, R. et al. [20] proposed a road friction coefficient estimation method that is effective when the vehicle has enough lateral excitation. Qi, Z. et al. [21] designed a Kalman Filter to estimate the longitudinal and lateral forces of each tire, as well as the derivatives of the two forces, using a 4 DOF(Degree of Freedom) vehicle model, and then the road friction coefficient was estimated according to the estimated tire lateral force. Compared with lateral force, self-aligning torque enters the nonlinear region earlier, so less lateral excitation is needed to estimate the road friction coefficient. Therefore, the relationship between the sideslip angle and self-aligning torque has been applied in many studies to obtain an accurate road friction coefficient. Luque, P. et al. [22] used a Kalman Filter to estimate longitudinal and lateral tire forces, and the self-aligning torque of the tire was calculated by a pretrained neural network. Then, the road friction coefficient was obtained by the relation curves between self-aligning torque and the sideslip angle in different road states. Matsuda, T. et al. [23] considered the road friction coefficient as a state and designed an EKF using a nonlinear 2 DOF single-track vehicle model, and self-aligning torque was measured to update the states. With varying road friction, Hsu, Y.-H.J. et al. [24] estimated the road friction coefficient from the relationships between (i) self-aligning torque and the tire trail and (ii) the tire trail and the sideslip angle. Ahn, C et al. [25] used a Kalman Filter to estimate the self-aligning torque of tires on the basis of the steering system and designed a nonlinear observer to estimate the road friction coefficient using self-aligning torque and a nonlinear vehicle model, and the stability and robustness of the nonlinear observer were proved. Shao, L. and Jin, C. [26] adopted a novel strategy to estimate the front axle lateral force. Then, combined with an indirect measurement based on total aligning torque estimation, a nonlinearadaptive observer was designed to estimate the road friction coefficient with guaranteed stability. The self-aligning torque of the front axle is coupled with the sideslip angle; so, to precisely calculate self-aligning torque, we must know the current sideslip angles of each tire. Therefore, an accurate sideslip angle contributes to improvement in the estimation accuracy of the road friction coefficient using self-aligning torque-based estimation methods. With the development of intelligent vehicles, besides conventional onboard sensors, such as steering wheel angle sensors, wheel speed sensors, and inertial measurement units, information from new sensors equipped on intelligent vehicles can also be used to estimate the vehicle state. Yoon, J. and Peng, H. [27] used velocity measurements from two GPS receivers to estimate the sideslip angle. To reduce the cost, they took advantage of the direction measurement using a magnetometer and proposed a sideslip angle estimation method that integrated a magnetometer with a GPS [28]. Wang, Y. et al. [29] proposed a combined vehicle and vision model to increase the robustness of the body-slip-angle estimation to uncertain vehicle parameters, and multi-rate and time-delay issues were explained. Furthermore, camera-aided estimation of the lateral state for the integrated control of automated vehicles was discussed in Reference [30]. 

Since new sensors equipped on intelligent vehicles facilitate the estimation of vehicle states, they could be useful for improving the accuracy of the results of road friction coefficient estimation. In this paper, we introduced vehicle lateral displacement, which is based on the relationship between road friction and the self-aligning torque of the front axle, to the framework of road friction coefficient estimation. On the one hand, lateral displacement information contributes to improvement in the estimation accuracy of the vehicle’s sideslip angle so that tire forces can be estimated more precisely. For intelligent vehicles, vehicle lateral displacement information can be obtained from cameras, GNSS (Global Navigation Satellite System) and maps, or V2X (Vehicle to Everything) systems. We acquired this information from a high-accuracy GNSS and a pre-established lane line map. On the other hand, compared with methods based on the relationship between road friction and the longitudinal or lateral forces of the tires, the self-aligning torque-based method requires fewer excitations, so the road friction coefficient can be estimated before the vehicle drives into critical conditions. We adjusted the tire brush model to fit the tire test data more accurately. Then, by integrating lateral displacement information, self-aligning torque measurement, lateral acceleration measurement, the tire model, and the vehicle model, we developed a nonlinear observer for road friction coefficient estimation. The stability of the observer was proved, and the observer’s robustness was analyzed. 

The main contributions of this paper are summarized as follows:

- A novel modified tire brush model based on tire test data is proposed. Compared with the traditional tire brush model, new mapping relationships between lateral tire force and the sideslip angle and between self-aligning torque and the sideslip angle are established, which can model tire forces and self-aligning torque more precisely. Further, the simple expression form of the modified tire model functions facilitates the proof of the non-linear observer’s stability.
- Lateral displacement information is introduced into the estimation system. Lateral displacement information can be obtained from new sensors equipped on intelligent vehicles, and it can be useful for accurate sideslip angle estimation, so that the road friction coefficient can be calculated more precisely.
- A non-linear observer for the road friction coefficient is proposed. The stability of the nonlinear observer is proved thorough the Lyapunov method, and the robustness is analyzed.

The remainder of this paper is organized as follows. In Section 2, the vehicle model and modified tire model are introduced. In Section 3, the nonlinear observer for road friction coefficient estimation is proposed, and its stability and robustness are analyzed. Section 4 presents experiments that were conducted to prove the proposed estimation method, and the experimental results are discussed. Finally, the conclusions are summarized in Section 5.

## 2. Vehicle and Tire Model

### 2.1. Vehicle Model

**Figure 1:**
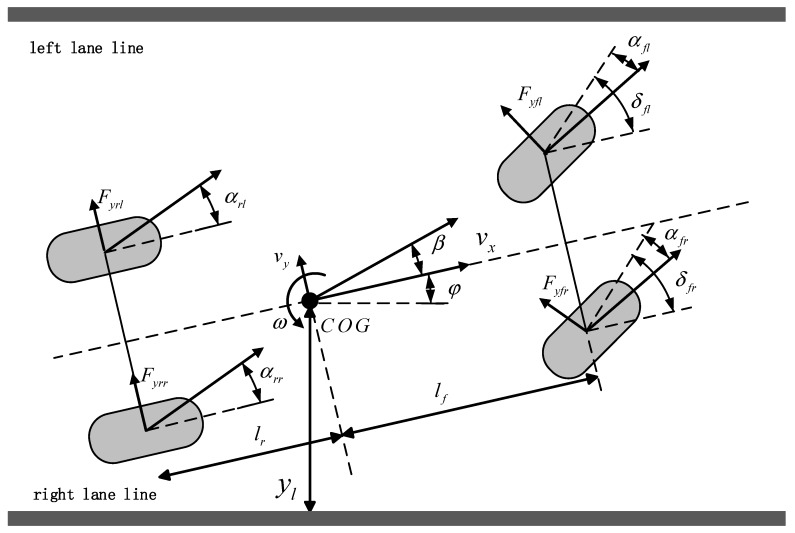
Vehicle model.

A nonlinear 2 DOF vehicle model is introduced to express the vehicle lateral dynamics, as shown in Figure 1. Both longitudinal and lateral load transfer are considered, and the dynamic model is expressed as:

(1) $$\dot{\omega }=\frac{l_{f}}{I_{z}}(F_{yfl}\cos\delta_{fl}+F_{yfr}\cos\delta_{fr})-\frac{l_{r}}{I_{z}}(F_{yrl}+F_{yrr})$$

(2) $$\dot{\beta }=\frac{a_{y}}{v_{x}}-\omega$$

where ω is the yaw rate; $l_{f}$ and $l_{r}$ are the distance between the COG (Center of Gravity) and the front and rear axles, respectively; $I_{z}$ is the vehicle yaw moment of inertia; $F_{yfl}$, $F_{yfr}$, $F_{yrf}$, and $F_{yrr}$ are the lateral forces of the front left tire, front right tire, rear left tire, and rear right tire, respectively; $\delta_{fl}$ and $\delta_{fr}$ are the steering angles of the front left wheel and front right wheel, respectively; β is the sideslip angle; $a_{y}$ is the lateral acceleration of the vehicle; $v_{x}$ is the longitudinal speed of the vehicle.

Lateral force $F_{y}$ is calculated by the tire model, and it corresponds to the road friction coefficient μ and sideslip angle α of each tire and vertical load $F_{z}$. The sideslip angles of tires are:

(3) $$\left\{ \begin{array}{lll} \alpha_{fl}=\frac{v_{y}+l_{f}\omega }{v_{x}-\frac{b}{2}\omega }-\delta_{fl} & & \alpha_{rl}=\frac{v_{y}-l_{r}\omega }{v_{x}-\frac{b}{2}\omega }\\ \alpha_{fr}=\frac{v_{y}+l_{f}\omega }{v_{x}+\frac{b}{2}\omega }-\delta_{fr} & & \alpha_{rr}=\frac{v_{y}-l_{r}\omega }{v_{x}+\frac{b}{2}\omega } \end{array}\right.$$

where $v_{y}$ is the lateral speed of the vehicle, and b is track base. Given the load transfer, the vertical load of each tire is:

(4) $$\left\{ \begin{array}{l} F_{zfl}=mg\frac{l_{r}}{2(l_{f}+l_{r})}-ma_{x}\frac{h}{2(l_{f}+l_{r})}-ma_{y}\frac{hl_{r}}{b(l_{f}+l_{r})}\\ F_{zfr}=mg\frac{l_{r}}{2(l_{f}+l_{r})}-ma_{x}\frac{h}{2(l_{f}+l_{r})}+ma_{y}\frac{hl_{r}}{b(l_{f}+l_{r})}\\ F_{zfl}=mg\frac{l_{r}}{2(l_{f}+l_{r})}+ma_{x}\frac{h}{2(l_{f}+l_{r})}+ma_{y}\frac{hl_{f}}{b(l_{f}+l_{r})}\\ F_{zfl}=mg\frac{l_{r}}{2(l_{f}+l_{r})}+ma_{x}\frac{h}{2(l_{f}+l_{r})}-ma_{y}\frac{hl_{f}}{b(l_{f}+l_{r})} \end{array}\right.$$

where $F_{zfl}$, $F_{zfr}$, $F_{zrf}$, and $F_{zrr}$ are the vertical forces of the front left tire, front right tire, rear left tire, and rear right tire, respectively; m is the vehicle mass; $a_{x}$ is longitudinal acceleration; h is the height of the COG.

### 2.2. Tire Model

A novel modified tire brush model is applied to describe lateral force $F_{y}$ and the self-aligning torque of the tire $M_{z}$. Compared with the traditional tire brush model, the proposed modified tire model has a simpler form and fits the tire test data better, so it is more convenient for road friction coefficient estimation during normal steering conditions. In the proposed modified tire model, the relationship between $\frac{\alpha }{F_{z}^{0.15}}$ and $\frac{F_{y}}{F_{z}^{0.81}}$ and the relationship between $\frac{\alpha }{F_{z}^{0.45}}$ and $\frac{M_{z}}{F_{z}^{1.85}}$ are established, as shown in Equations (5) and (6), respectively. With the new mapping relationships, the tire model can describe the variation in lateral force and self-alignment with different vertical loads more precisely.

(5) $$\frac{F_{y}}{F_{z}^{0.81}}=-\mathrm{sign}\left(\alpha \right)•3d_{1}\mu \frac{c_{1}}{3\mu }\left(\frac{\left|\alpha \right|}{F_{z}^{0.15}}\right)\left\{1-\frac{c_{1}}{3\mu }\left(\frac{\left|\alpha \right|}{F_{z}^{0.15}}\right)+\frac{1}{3}{\left[\frac{c_{1}}{3\mu }\left(\frac{\left|\alpha \right|}{F_{z}^{0.15}}\right)\right]}^{2}\right\}$$

(6) $$\frac{M_{z}}{F_{z}^{1.85}}=\mathrm{sign}\left(\alpha \right)•\frac{d_{2}}{2}\mu \frac{c_{2}}{3\mu }\left(\frac{\left|\alpha \right|}{F_{z}^{0.45}}\right){\left[1-\frac{c_{2}}{3\mu }\left(\frac{\left|\alpha \right|}{F_{z}^{0.45}}\right)\right]}^{3}$$

where $d_{1}$, $d_{2}$, $c_{1}$, and $c_{2}$ are parameters, and μ is the road friction coefficient. 

**Figure 2:**
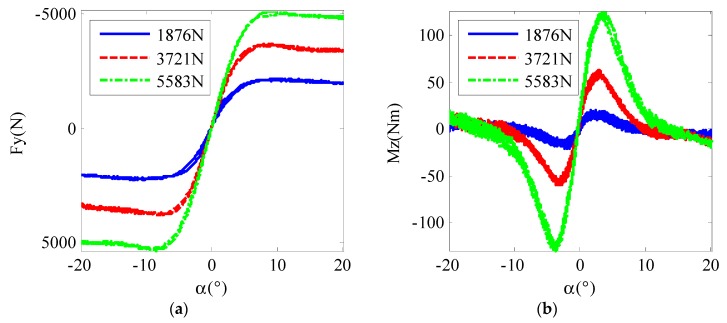
Row tire test data: (**a**) lateral force; (**b**) self-aligning torque.

**Figure 3:**
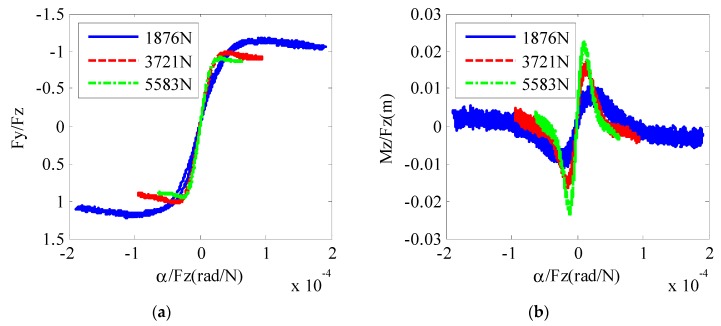
Normalization of tire test data with the original tire brush model: (**a**) lateral force; (**b**) self-aligning torque.

**Figure 4:**
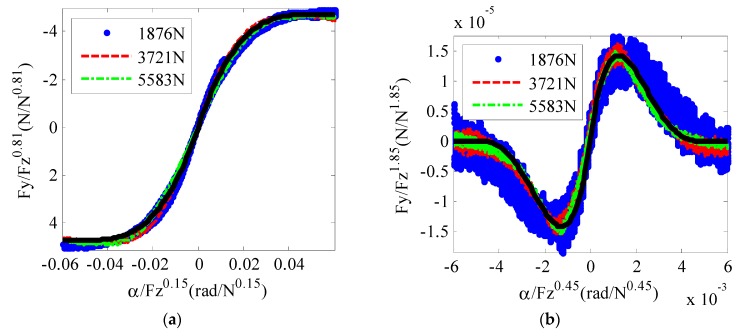
Normalization of tire test data with the modified tire brush model: (**a**) lateral force; (**b**) self-aligning torque.

Tire tests were conducted on a tire test bench to verify the proposed tire model. The raw test data are shown in Figure 2. If we normalize the lateral tire force with the vertical load using the traditional tire brush model, the curves for different tire loads do not line up very well, as shown in Figure 3, which means that $F_{y}$ and $M_{z}$ are not directly correlative with $F_{z}$. With the new relationship proposed in the modified tire brush model, the test data are normalized, as shown in Figure 4. For lateral tire force, Figure 4a reveals that the relationships between $\frac{\alpha }{F_{z}^{0.15}}$ and $\frac{F_{y}}{F_{z}^{0.81}}$ calculated by the modified tire model are nearly the same for different tire loads. Similarly, the test results in Figure 4b show that the relationships between $\frac{\alpha }{F_{z}^{0.45}}$ and $\frac{M_{z}}{F_{z}^{1.85}}$ are the same for different vertical loads. Therefore, the proposed modified tire brush model can calculate the lateral tire force more precisely with tire load variation.

The tire test data in Figure 4 prove the function form of the modified tire model, so the next step is to use the test data normalized by the vertical load to fit the tire model function. The fitting results are shown as the black line in Figure 4, and the proposed modified tire model can be expressed as:

(7) $$F_{y}(\alpha ,F_{z},\mu )=-\mathrm{sign}\left(\alpha \right)•3d_{1}\mu F_{z}^{0.81}\frac{c_{1}}{3\mu }\left(\frac{\left|\alpha \right|}{F_{z}^{0.15}}\right)\left\{1-\frac{c_{1}}{3\mu }\left(\frac{\left|\alpha \right|}{F_{z}^{0.15}}\right)+\frac{1}{3}{\left[\frac{c_{1}}{3\mu }\left(\frac{\left|\alpha \right|}{F_{z}^{0.15}}\right)\right]}^{2}\right\}$$

(8) $$M_{z}(\alpha ,F_{z},\mu )=\mathrm{sign}\left(\alpha \right)•\frac{d_{2}}{2}\mu F_{z}^{1.85}\frac{c_{2}}{3\mu }\left(\frac{\left|\alpha \right|}{F_{z}^{0.45}}\right){\left[1-\frac{c_{2}}{3\mu }\left(\frac{\left|\alpha \right|}{F_{z}^{0.45}}\right)\right]}^{3}$$

## 3. Nonlinear Observer Design for Road Friction Coefficient Estimation

### 3.1. NonlinearObserver Design

Assuming that the road friction coefficient μ is piecewise constant and using the vehicle dynamic model (2) we have:

(9) $$\dot{\mu }=0$$

(10) $${\dot{v}}_{y}=a_{y}\left(\mu ,v_{y},F_{z}\right)-\omega v_{x}$$

where $a_{y}\left(\mu ,v_{y},F_{z}\right)$ is lateral acceleration, which is:

(11) $$\begin{array}{cc} a_{y}\left(\mu ,v_{y},F_{z}\right) & =\frac{1}{m}\left(F_{yfl}\cos\delta_{fl}+F_{yfr}\cos\delta_{fr}+F_{yrl}+F_{yrr}\right)\\ & =\frac{1}{m}[F_{y}\left(\mu ,\alpha_{fl},F_{zfl}\right)\cos\delta_{fl}+F_{y}\left(\mu ,\alpha_{fr},F_{zfr}\right)\cos\delta_{fr}+F_{y}\left(\mu ,\alpha_{rl},F_{zrl}\right)+F_{y}\left(\mu ,\alpha_{rr},F_{zrr}\right)] \end{array}$$

Since the lane line map information is available, we can measure (i) the distance between the COG of the vehicle and the lane line $y_{l}$ and (ii) the angle between the lane line and vehicle heading φ. $y_{l}$ and φ can either be obtained by a camera installed on the vehicle or calculated through the location information from a GNSS receiver and lane map, which is a priori knowledge. The lateral displacement can also be obtained through V2X technology; for example, through the UWB (Ultra-Wideband) localization technique, the distance between the vehicle and infrastructure along the road can be calculated. According to the kinematic relationships of vehicle motion, the dynamics of the distance between the COG and the lane line can be expressed as:

(12) $${\dot{y}}_{l}=v_{x}\sin\varphi +v_{y}\cos\varphi$$

From the system defined by Equations (10)–(12), the corresponding nonlinear observer isdesigned as:

(13) $$\dot{\hat{\mu }}=k_{1}\mathrm{sign}(f_{1}{)[M}_{k}-f_{k}(\hat{\mu },\hat{\alpha },F_{z})]+k_{2}\mathrm{sign}(f_{2})[a_{y}-a_{y}(\hat{\mu },\hat{\alpha },F_{z})]$$

(14) $${\dot{\hat{y}}}_{l}=v_{x}\sin\varphi +{\hat{v}}_{y}\cos\varphi +k_{3}\left(y_{l}-{\hat{y}}_{l}\right)$$

(15) $${\dot{\hat{v}}}_{y}=a_{y}\left(\hat{\mu },{\hat{v}}_{y},F_{Z}\right)-rv_{x}+k_{4}\left(y_{l}-{\hat{y}}_{l}\right)+k_{5}\left[a_{y}-a_{y}\left(\hat{\mu },{\hat{v}}_{y},F_{Z}\right)\right]+k_{6}\int_{0}^{t}\left[a_{y}-a_{y}\left(\hat{\mu },{\hat{v}}_{y},F_{Z}\right)\right]dt$$

where the superscript ^ denotes an estimated value; $k_{1}$, $k_{2}$, $k_{3}$, $k_{4}$, $k_{5}$, and $k_{6}$ are parameters; $k_{1}$, $k_{2}$, $k_{3}$ and $k_{6}$ are positive, $k_{4}=\cos\varphi$. From Equation (13)$f_{1}$ is defined as:

(16) $$\{\begin{array}{cc} f_{1}>0 & \alpha_{f}>0,{\hat{\alpha }}_{f}>0\\ f_{1}<0 & \alpha_{f}<0,{\hat{\alpha }}_{f}<0\\ f_{1}=0 & else \end{array}$$

and $f_{2}$ is defined as:

(17) $$\{\begin{array}{cc} f_{2}<0 & \alpha_{f}>0,{\hat{\alpha }}_{f}>0,\alpha_{r}>0,{\hat{\alpha }}_{r}>0\\ f_{2}>0 & \alpha_{f}<0,{\hat{\alpha }}_{f}<0,\alpha_{r}<0,{\hat{\alpha }}_{r}<0\\ f_{2}=0 & else \end{array}$$

$M_{k}$ is the self-aligning torque at the kingpin, and it can be calculated as:

(18) $$M_{k}=F_{kl}L_{l}(\delta_{fl})-F_{kr}L_{r}(\delta_{fr})$$

where $F_{kl}$ and $F_{kr}$ are tie rod forces on the left and right sides, respectively. $L_{l}(\delta_{fl})$ and $L_{r}(\delta_{lr})$ are the distances from the steering rods to the kingpin on the left and right sides, respectively. It has to be mentioned that, in our approach, $F_{kl}$ and $F_{kr}$ are measured by tension and compression force sensors installed at the left and right tie rods. This measurement can be provided by the steer-by-wire system of intelligent vehicles. It can also be estimated by the steering system if the algorithm of the EPS (Electric Power Steering) system is available. $f_{k}\left(\hat{\mu },{\hat{v}}_{y},F_{z}\right)$ is the self-aligning torque at the kingpin estimated by the wheel steering model and tire model, which can be expressed as:

(19) $$\begin{array}{cc} f_{k}\left(\mu ,v_{y},F_{z}\right) & =M_{zfl}+M_{zfr}+l_{ml}F_{yfl}+l_{mr}F_{yfr}\\ & =M_{z}\left(\mu ,\alpha_{fl},F_{zfl}\right)+M_{z}\left(\mu ,\alpha_{fr},F_{zfr}\right)+l_{ml}F_{y}\left(\mu ,\alpha_{fl},F_{zfl}\right)+l_{mr}F_{y}\left(\mu ,\alpha_{fr},F_{zfr}\right) \end{array}$$

where $l_{ml}$ and $l_{mr}$ are the mechanical trails of the left and right front tires, respectively.

### 3.2. Stability Analysis

According to the system dynamics in (9), (10), (12) and nonlinear observer in (13), (14), (15), the corresponding error dynamics is:

(20) $$\dot{\tilde{\mu }}=-k_{1}\mathrm{sign}(f_{1})\left[f_{k}\left(\mu ,v_{y},F_{z}\right)-f_{k}\left(\hat{\mu },{\hat{v}}_{y},F_{z}\right)\right]-k_{2}\mathrm{sign}(f_{2})\left[a_{y}\left(\mu ,v_{y},F_{z}\right)-a_{y}\left(\hat{\mu },{\hat{v}}_{y},F_{z}\right)\right]$$

(21) $${\dot{\tilde{y}}}_{l}=-k_{3}{\tilde{y}}_{l}+{\tilde{v}}_{y}\cos\varphi$$

(22) $$\begin{array}{cc} {\dot{\tilde{v}}}_{y} & =\left(1-k_{5}\right)\left[a_{y}\left(\mu ,v_{y},F_{z}\right)-a_{y}\left(\hat{\mu },{\hat{v}}_{y},F_{z}\right)\right]-k_{6}\int_{0}^{t}\left\{\left[a_{y}\left(\mu ,v_{y},F_{z}\right)-rv_{x}\right]-\left[{\hat{a}}_{y}\left(\hat{\mu },{\hat{v}}_{y},F_{z}\right)-rv_{x}\right]\right\}dt-k_{4}{\tilde{y}}_{l}\\ & =-k_{6}{\tilde{v}}_{y}-\left(k_{5}-1\right)\left[a_{y}\left(\mu ,v_{y},F_{z}\right)-a_{y}\left(\hat{\mu },{\hat{v}}_{y},F_{z}\right)\right]-k_{4}{\tilde{y}}_{l} \end{array}$$

where the superscript ~denotes the error between the estimated value and the real value.

The Lyapunov function is defined as:

(23) $$V=\frac{1}{2}{\tilde{\mu }}^{2}+\frac{1}{2}{\tilde{y}}_{l}^{2}+\frac{1}{2}{\tilde{v}}_{y}^{2}$$

Then, we have:

(24) $$\begin{array}{c} \dot{V}=\tilde{\mu }\left\{-k_{1}\mathrm{sign}(f_{1})\left[f_{k}\left(\mu ,v_{y},F_{z}\right)-f_{k}\left(\hat{\mu },{\hat{v}}_{y},F_{z}\right)\right]-k_{2}\mathrm{sign}(f_{2})\left[a_{y}\left(\mu ,v_{y},F_{z}\right)-a_{y}\left(\hat{\mu },{\hat{v}}_{y},F_{z}\right)\right]\right\}\\ +{\tilde{y}}_{l}\left\{-k_{3}{\tilde{y}}_{l}+\left(\cos\varphi \right){\tilde{v}}_{y}\right\}+{\tilde{v}}_{y}\left\{-k_{6}{\tilde{v}}_{y}-\left(k_{5}-1\right)\left[a_{y}\left(\mu ,v_{y},F_{z}\right)-a_{y}\left(\hat{\mu },{\hat{v}}_{y},F_{z}\right)\right]-k_{4}{\tilde{y}}_{l}\right\} \end{array}$$

Using the mean value theorem, we have:

(25) $$f_{k}\left(\mu ,v_{y},F_{z}\right)-f_{k}\left(\hat{\mu },{\hat{v}}_{y},F_{z}\right)=\frac{\partial {\bar{f}}_{k}}{\partial \mu }\tilde{\mu }+\frac{\partial {\bar{f}}_{k}}{\partial v_{y}}{\tilde{v}}_{y}$$

(26) $$a_{y}\left(\mu ,v_{y},F_{z}\right)-a_{y}\left(\hat{\mu },{\hat{v}}_{y},F_{z}\right)=\frac{\partial {\bar{a}}_{y}}{\partial \mu }\tilde{\mu }+\frac{\partial {\bar{a}}_{y}}{\partial v_{y}}{\tilde{v}}_{y}$$

where

(27) $$\left\{ \begin{array}{l} \frac{\partial {\bar{f}}_{k}}{\partial \mu }=\frac{\partial f_{k}}{\partial \mu }\left(\bar{\mu },{\bar{v}}_{y}\right)\\ \frac{\partial {\bar{f}}_{k}}{\partial v_{y}}=\frac{\partial f_{k}}{\partial v_{y}}\left(\bar{\mu },{\bar{v}}_{y}\right)\\ \frac{\partial {\bar{a}}_{y}}{\partial \mu }=\frac{\partial a_{y}}{\partial \mu }\left(\bar{\mu },{\bar{v}}_{y}\right)\\ \frac{\partial {\bar{a}}_{y}}{\partial v_{y}}=\frac{\partial a_{y}}{\partial v_{y}}\left(\bar{\mu },{\bar{v}}_{y}\right) \end{array}\right.$$

In Equation (27) $\bar{\mu }$ is a median between μ and $\hat{\mu }$; ${\bar{v}}_{y}$ is a median between $v_{y}$ and ${\hat{v}}_{y}$. Substituting (25), (26) and (27) into (24) we have:

(28) $$\begin{array}{cc} \dot{V}= & -\left[k_{1}\mathrm{sign}(f_{1})\frac{\partial {\bar{f}}_{k}}{\partial \mu }+k_{2}\mathrm{sign}(f_{2})\frac{\partial {\bar{a}}_{y}}{\partial \mu }\right]{\tilde{\mu }}^{2}-k_{3}{\tilde{y}}_{l}^{2}-\left[\left(k_{5}-1\right)\frac{\partial {\bar{a}}_{y}}{\partial v_{y}}+k_{6}\right]{\tilde{v}}_{y}^{2}\\ & -\left[k_{1}\mathrm{sign}(f_{1})\frac{\partial {\bar{f}}_{k}}{\partial v_{y}}+k_{2}\mathrm{sign}(f_{2})\frac{\partial {\bar{a}}_{y}}{\partial v_{y}}+\left(k_{5}-1\right)\frac{\partial {\bar{a}}_{y}}{\partial \mu }\right]\tilde{\mu }{\tilde{v}}_{y}+\left(-k_{4}+\cos\varphi \right){\tilde{y}}_{l}{\tilde{v}}_{y}\\ = & -\left[\begin{array}{ccc} \tilde{\mu } & {\tilde{y}}_{l} & {\tilde{v}}_{y} \end{array}\right]A\left[\begin{array}{c} \tilde{\mu }\\ {\tilde{y}}_{l}\\ {\tilde{v}}_{y} \end{array}\right] \end{array}$$

where

(29) $$A=\left[\begin{array}{ccc} A_{11} & 0 & A_{13}\\ 0 & k_{3} & A_{23}\\ A_{31} & A_{32} & A_{33} \end{array}\right]$$

(30) $$A_{11}=k_{1}\mathrm{sign}(f_{1})\frac{\partial {\bar{f}}_{k}}{\partial \mu }+k_{2}\mathrm{sign}(f_{2})\frac{\partial {\bar{a}}_{y}}{\partial \mu }$$

(31) $$A_{13}=A_{31}=\frac{1}{2}k_{1}\mathrm{sign}(f_{1})\frac{\partial {\bar{f}}_{k}}{\partial v_{y}}+\frac{1}{2}k_{2}\mathrm{sign}(f_{2})\frac{\partial {\bar{a}}_{y}}{\partial v_{y}}+\frac{1}{2}\left(k_{5}-1\right)\frac{\partial {\bar{a}}_{y}}{\partial \mu }$$

(32) $$A_{23}=A_{32}=-\frac{1}{2}k_{4}+\frac{1}{2}\cos\varphi$$

(33) $$A_{33}=\left(k_{5}-1\right)\frac{\partial {\bar{a}}_{y}}{\partial v_{y}}+k_{6}$$

If A is a positive definite matrix, then $\dot{V}<0$ holds, which means that the estimation system is stable, and the estimation error will converge to zero as time t→∞. To ensure that A is a positive definite matrix, all sequential principal minors of A need to be positive. According to the modified tire model in (7) and (8) and the symbolic function defined in (16) and (17) it can be deduced that:

(34) $$\mathrm{sign}(f_{1})\frac{\partial {\bar{f}}_{k}}{\partial \mu }\geq 0$$

(35) $$\mathrm{sign}(f_{2})\frac{\partial {\bar{a}}_{y}}{\partial \mu }\geq 0$$

Therefore, if we choose $k_{1}$, $k_{2}$, and $k_{3}$ as positive constants, the first-order and second-order sequential principal minors of A are positive. If we choose $k_{4}=\cos\varphi$, then the third-order sequential principal minor of A is:

(36) $$\begin{array}{cc} \left|A\right|= & k_{3}\left[k_{1}\mathrm{sign}(f_{1})\frac{\partial {\bar{f}}_{k}}{\partial \mu }+k_{2}\mathrm{sign}(f_{2})\frac{\partial {\bar{a}}_{y}}{\partial \mu }\right]\left[\left(k_{5}-1\right)\frac{\partial {\bar{a}}_{y}}{\partial v_{y}}+k_{6}\right]\\ & -\frac{k_{3}}{4}{\left[k_{1}\mathrm{sign}(f_{1})\frac{\partial {\bar{f}}_{k}}{\partial v_{y}}+k_{2}\mathrm{sign}(f_{2})\frac{\partial {\bar{a}}_{y}}{\partial v_{y}}+\left(k_{5}-1\right)\frac{\partial {\bar{a}}_{y}}{\partial \mu }\right]}^{2} \end{array}$$

Since $\frac{\partial {\bar{f}}_{k}}{\partial v_{y}}$, $\frac{\partial {\bar{a}}_{y}}{\partial v_{y}}$, and $\frac{\partial {\bar{a}}_{y}}{\partial \mu }$ are bounded according to the modified tire model, there exists a parameter $k_{5}$ such that:

(37) $${\left[k_{1}\mathrm{sign}(f_{1})\frac{\partial {\bar{f}}_{k}}{\partial v_{y}}+k_{2}\mathrm{sign}(f_{2})\frac{\partial {\bar{a}}_{y}}{\partial v_{y}}+\left(k_{5}-1\right)\frac{\partial {\bar{a}}_{y}}{\partial \mu }\right]}^{2}={\left[A_{11}+\left(k_{5}-1\right)\frac{\partial {\bar{a}}_{y}}{\partial \mu }\right]}^{2}=0$$

If the chosen value of $k_{6}$ is large enough, then |A|>0 holds. Therefore, $\dot{V}<0$ holds, and it can be deduced that:

(38) $$\left[\begin{array}{c} \tilde{\mu }\\ {\tilde{y}}_{l}\\ {\tilde{v}}_{y} \end{array}\right]\to \left[\begin{array}{c} 0\\ 0\\ 0 \end{array}\right]\;\mathrm{as}\;t\to \infty$$

and the estimation error can converge to zero exponentially.

### 3.3. Robustness Analysis

The uncertainties of the tire and vehicle models or measurements from sensors introduce perturbance to the system. It is necessary to analyze the performance of the estimator with bounded external excitation. According to the error dynamics of the system in (20), (21) and (22) without external inputs, the error dynamics of the system with inputs can be expressed as

(39) $$\dot{\tilde{\mu }}=-k_{1}\mathrm{sign}(f_{1})\left(\frac{\partial {\bar{f}}_{k}}{\partial \mu }\tilde{\mu }+\frac{\partial {\bar{f}}_{k}}{\partial v_{y}}{\tilde{v}}_{y}\right)-k_{2}\mathrm{sign}(f_{2})\left(\frac{\partial {\bar{a}}_{y}}{\partial \mu }\tilde{\mu }+\frac{\partial {\bar{a}}_{y}}{\partial v_{y}}{\tilde{v}}_{y}\right)+u_{1}$$

(40) $${\dot{\tilde{y}}}_{l}=-k_{3}{\tilde{y}}_{l}+{\tilde{v}}_{y}\cos\varphi +u_{2}$$

(41) $${\dot{\tilde{v}}}_{y}=-k_{6}{\tilde{v}}_{y}-\left(k_{5}-1\right)\left(\frac{\partial {\bar{a}}_{y}}{\partial \mu }\tilde{\mu }+\frac{\partial {\bar{a}}_{y}}{\partial v_{y}}{\tilde{v}}_{y}\right)-k_{4}{\tilde{y}}_{l}+u_{3}$$

where $u_{1}$, $u_{2}$, and $u_{3}$ are external bounded inputs. $u_{1}$ includes the uncertainties of the tire model and the uncertainties in the estimation results of $v_{y}$. $u_{2}$ includes the uncertainties in the measurements of lateral distance between the COG and the lane line and uncertainties in the estimation results of $v_{y}$.$u_{3}$ includes the uncertainties in lateral distance measurements and uncertainties in the estimation results of μ.

The Lyapunov function is chosen and defined in Equation (23) thus, according to (39), (40), and (41) we have:

(42) $$\begin{array}{cc} \dot{V}= & \tilde{\mu }\left\{-k_{1}\mathrm{sign}(f_{1})\left(\frac{\partial {\bar{f}}_{k}}{\partial \mu }\tilde{\mu }+\frac{\partial {\bar{f}}_{k}}{\partial v_{y}}{\tilde{v}}_{y}\right)-k_{2}\mathrm{sign}(f_{2})\left(\frac{\partial {\bar{a}}_{y}}{\partial \mu }\tilde{\mu }+\frac{\partial {\bar{a}}_{y}}{\partial v_{y}}{\tilde{v}}_{y}\right)+u_{1}\right\}\\ & +{\tilde{y}}_{l}\left\{-k_{3}{\tilde{y}}_{l}+\left(\cos\varphi \right){\tilde{v}}_{y}+u_{2}\right\}+{\tilde{v}}_{y}\left\{-k_{6}{\tilde{v}}_{y}-\left(k_{5}-1\right)\left(\frac{\partial {\bar{a}}_{y}}{\partial \mu }\tilde{\mu }+\frac{\partial {\bar{a}}_{y}}{\partial v_{y}}{\tilde{v}}_{y}\right)-k_{4}{\tilde{y}}_{l}+u_{3}\right\}\\ = & -A_{11}{\tilde{\mu }}^{2}-k_{3}{\tilde{y}}_{l}{}^{2}-A_{33}{\tilde{v}}_{y}{}^{2}-\left[A_{11}+\left(k_{5}-1\right)\frac{\partial {\bar{a}}_{y}}{\partial \mu }\right]\tilde{\mu }{\tilde{v}}_{y}+\left(\cos\varphi -k_{4}\right){\tilde{v}}_{y}{\tilde{y}}_{l}+\tilde{\mu }u_{1}+{\tilde{y}}_{l}u_{2}+{\tilde{v}}_{y}u_{3} \end{array}$$

Since $k_{4}=\cos\varphi$, substituting (37) into (42) we have

(43) $$\begin{array}{cc} \dot{V}= & -A_{11}{\tilde{\mu }}^{2}-k_{3}{\tilde{y}}_{l}{}^{2}-A_{33}{\tilde{v}}_{y}{}^{2}+\tilde{\mu }u_{1}+{\tilde{y}}_{l}u_{2}+{\tilde{v}}_{y}u_{3}\\ \leq & -A_{11}{\left|\tilde{\mu }\right|}^{2}-k_{3}{\left|{\tilde{y}}_{l}\right|}^{2}-A_{33}{\left|{\tilde{v}}_{y}\right|}^{2}+\left|\tilde{\mu }\right|\left|u_{1}\right|+\left|{\tilde{y}}_{l}\right|\left|u_{2}\right|+\left|{\tilde{v}}_{y}\right|\left|u_{3}\right|\\ = & \left[-A_{11}(1-\theta_{1}){\left|\tilde{\mu }\right|}^{2}-A_{11}\theta_{1}{\left|\tilde{\mu }\right|}^{2}+\left|\tilde{\mu }\right|\left|u_{1}\right|\right]+\left[-k_{3}(1-\theta_{2}){\left|{\tilde{y}}_{l}\right|}^{2}-k_{3}\theta_{2}{\left|{\tilde{y}}_{l}\right|}^{2}+\left|{\tilde{y}}_{l}\right|\left|u_{2}\right|\right]\\ & +\left[-A_{33}(1-\theta_{3}){\left|{\tilde{v}}_{y}\right|}^{2}-A_{11}\theta_{1}{\left|{\tilde{v}}_{y}\right|}^{2}+\left|{\tilde{v}}_{y}\right|\left|u_{3}\right|\right]\\ = & -A_{11}(1-\theta_{1}){\left|\tilde{\mu }\right|}^{2}-k_{3}(1-\theta_{2}){\left|{\tilde{y}}_{l}\right|}^{2}-A_{33}(1-\theta_{3}){\left|{\tilde{v}}_{y}\right|}^{2}\\ & -\left(A_{11}\theta_{1}{\left|\tilde{\mu }\right|}^{2}-\left|\tilde{\mu }\right|\left|u_{1}\right|\right)-\left(k_{3}\theta_{2}{\left|{\tilde{y}}_{l}\right|}^{2}-\left|{\tilde{y}}_{l}\right|\left|u_{2}\right|\right)-\left(A_{11}\theta_{1}{\left|{\tilde{v}}_{y}\right|}^{2}-\left|{\tilde{v}}_{y}\right|\left|u_{3}\right|\right) \end{array}$$

where $0<\theta_{1}<1$, $0<\theta_{2}<1$, and $0<\theta_{3}<1$. Therefore, if the bounded inputs satisfy:

(44) $$\left\{ \begin{array}{l} \left|u_{1}\right|<A_{11}\theta_{1}\\ \left|u_{2}\right|<k_{3}\theta_{2}\\ \left|u_{3}\right|<A_{33}\theta_{3} \end{array}\right.$$

then we have:

(45) $$\dot{V}\leq -A_{11}(1-\theta_{1}){\left|\tilde{\mu }\right|}^{2}-k_{3}(1-\theta_{2}){\left|{\tilde{y}}_{l}\right|}^{2}-A_{33}(1-\theta_{3}){\left|{\tilde{v}}_{y}\right|}^{2}$$

If we define:

(46) $$A_{11}(1-\theta_{1}){\left|\tilde{\mu }\right|}^{2}+k_{3}(1-\theta_{2}){\left|{\tilde{y}}_{l}\right|}^{2}+A_{33}(1-\theta_{3}){\left|{\tilde{v}}_{y}\right|}^{2}=W\left(x\right)$$

where $x={\left[\begin{array}{ccc} \tilde{\mu } & {\tilde{y}}_{l} & {\tilde{v}}_{y} \end{array}\right]}^{T}$, then inequalities (47) (48) hold:

(47) $$\frac{1}{4}{\left\|x\right\|}^{2}\leq V\leq {\left\|x\right\|}^{2}$$

(48) $$\frac{\partial V}{\partial t}+\frac{\partial V}{\partial x}\dot{x}\leq -W\left(x\right).$$

By applying Theorem 4.19 from [31], we can reason that the system expressed in (39), (40), and (41) is input-state stable; thus, if the estimation system is interfered by bounded inputs, then the system will still stay stable.

## 4. Experimental Validation

Tests based on an electric vehicle were conducted to verify the proposed road friction coefficient estimation algorithm. The experimental setup and results are discussed in this section.

### 4.1. Experimental Setup

#### 4.1.1. Test Vehicle

**Figure 5:**
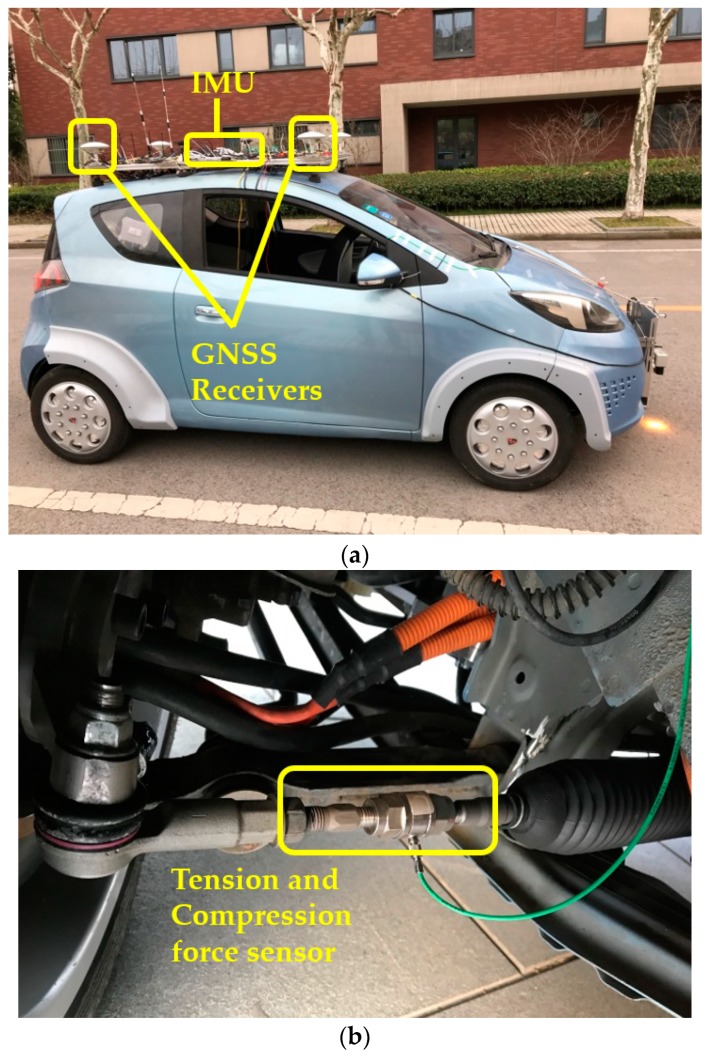
Test vehicle implementation: (**a**) test vehicle; (**b**) steering tie rod with a tension and compression force sensor.

The test vehicle is an electric vehicle and shown in Figure 5a, and the vehicle parameters are listed in Table 1. Information about the wheel speed and steering wheel angle was obtained through CAN-Bus. The GNSS receiver is Novatel 718D, which provides the absolute position and heading angle of the vehicle. It is necessary to point out that the lane lines were modeled in advance in the navigation coordinates so that the distance between the vehicle and lane line could be calculated in real time. ADIS16495 is the IMU (Inertial Measurement Unit), which measures the acceleration and angular velocities. The angle between the vehicle heading and lane line can be calculated by integrating the yaw rate of the vehicle. The steering tie rods are cut off and two Kistler tension and compression force sensors 9321B are installed on the left and right tie rods, respectively, as shown in Figure 5b. The tension and compression force sensors measure the force at the tie rods so that the self-aligning torque of the wheel can be measured indirectly.

#### 4.1.2. Test Road

**Figure 6:**
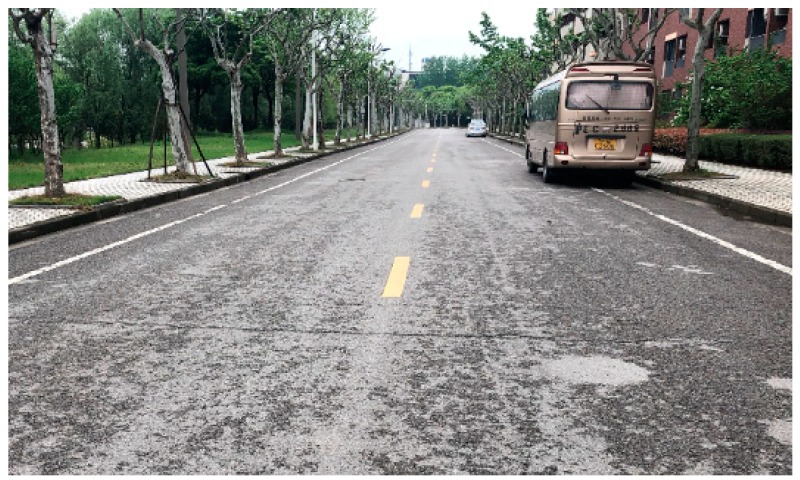
Test road.

To verify the proposed road friction coefficient estimation algorithm, slalom tests and DLC (Double Line Change) tests were conducted on the road shown in Figure 6. The white lane lines shown in Figure 6 were mapped in advance. According to a large number of emergency braking experiments, the real road friction coefficient is considered to be around 0.8.

### 4.2. Experimental Results and Analysis

#### 4.2.1. Slalom Test

**Figure 7:**
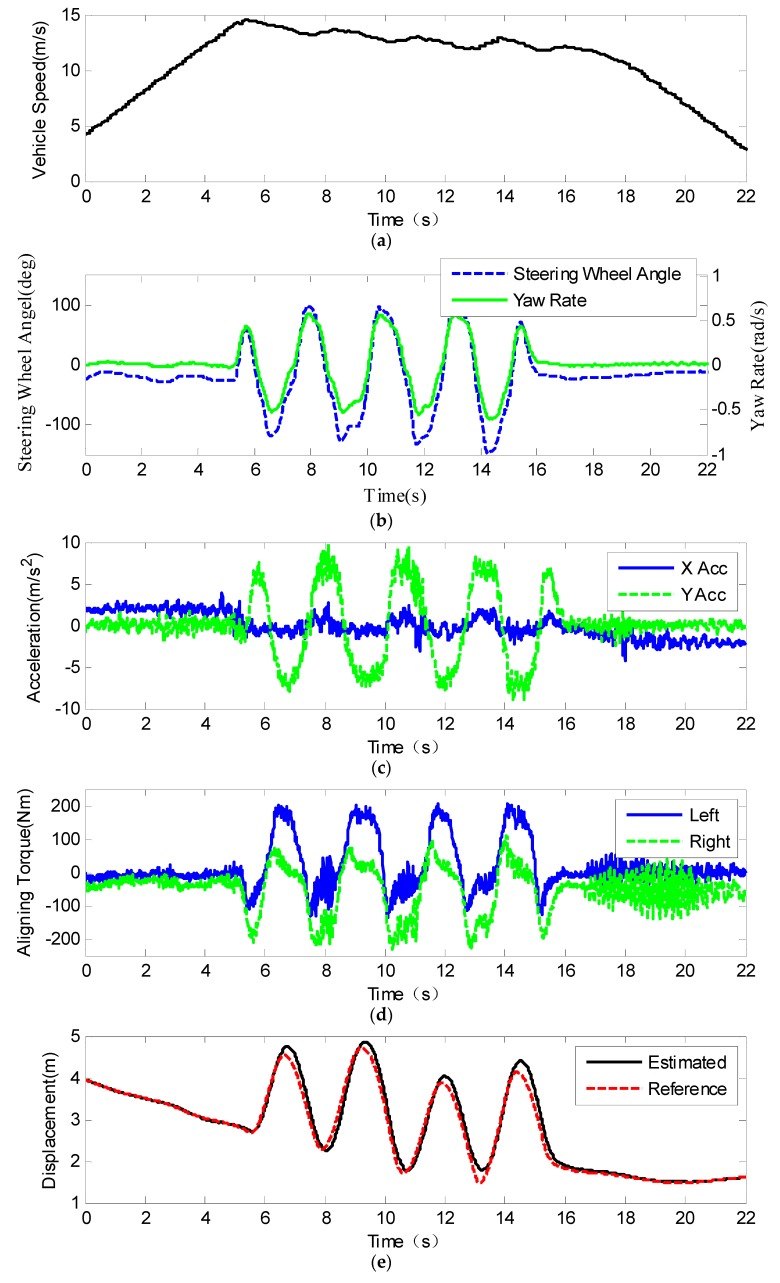

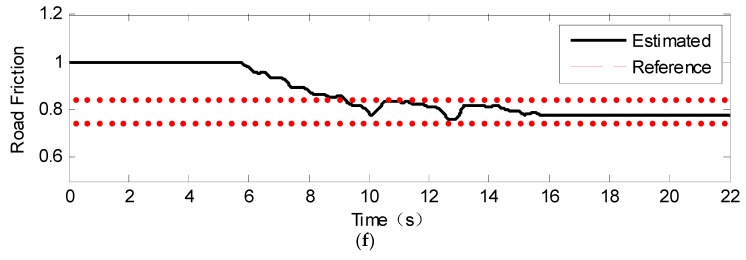
Results of the slalom test: (**a**) vehicle speed; (**b**) steering wheel angle and yaw rate; (**c**) longitudinal and lateral acceleration; (**d**) self-aligning torque at the kingpin; (**e**) distance between the vehicle and the left lane line; (**f**) road friction coefficient.

**Figure 8:**
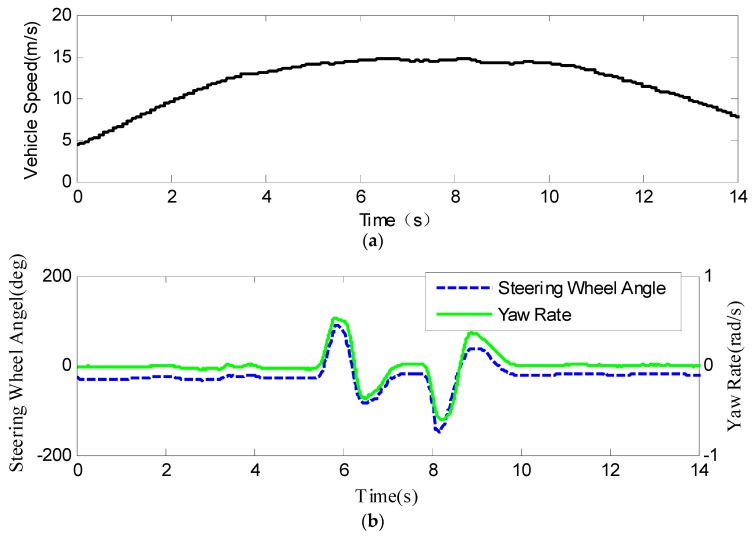

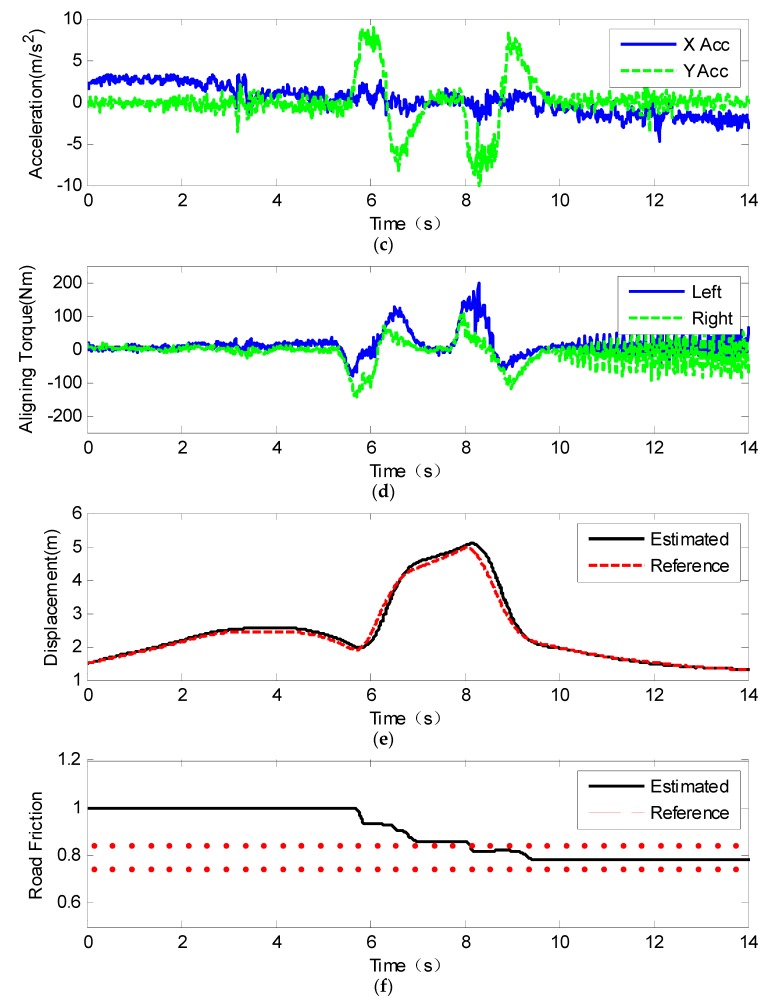
Results of the DLC (Double Line Change) test: (**a**) vehicle speed; (**b**) steering wheel angle and yaw rate; (**c**) longitudinal and lateral acceleration; (**d**) self-aligning torque at the kingpin; (**e**) distance between the vehicle and the left lane line; (**f**) road friction coefficient.

Slalom test results are shown in Figure 7. Figure 7a shows the vehicle speed measured by the GNSS receiver. The blue line and green line show the steering wheel angle and yaw rate during the test, respectively. Figure 7c shows the accelerations recorded by the IMU, and we can see that the maximum lateral acceleration reaches 8 $ms^{-2}$, which means that the vehicle has enough lateral excitation. Figure 7d shows the estimated self-aligning torque at the kingpin according to the tension and compression force sensors installed at the steering tie rods. Figure 7e shows the estimated lateral distance between the COG of the vehicle and the lane line. The reference is calculated by the absolute position and the lane line map. The road friction coefficient estimation result is shown in Figure 7f. Since the road friction coefficient is related not only to the road surface but also to the tires, an absolutely precise road friction coefficient could not be obtained. From braking tests, we know that the real friction coefficient is about 0.8; therefore, we set 0.75–0.85 as the reference region. The initial road friction coefficient is set at 1. From Figure 8f, we can see that at around 9 s, the estimated road coefficient converges to the reference region, and the convergence time was about 3 s with continuous lateral excitation. After 9 s, the estimated value remains within the reference region, although there is a slight fluctuation, which means that the nonlinear estimator performs well. If the reference value of the road friction is considered as 0.8, then the estimation accuracy was about 97.2%. From 16 s onward, the vehicle drives straightly, and the road friction coefficient estimation algorithm stops without lateral excitation.

#### 4.2.2. DLC Test

Figure 8 shows the experimental results of DLC maneuvering. Figure 8a shows the vehicle speed during the test, and in Figure 8b represents the steering wheel angle and yaw rate of the vehicle by the blue line and green line, respectively. Figure 8c shows the longitudinal and lateral accelerations of the vehicle, and the maximum lateral acceleration is between 8 and 9 $ms^{-2}$. The estimated aligning torques of the left and right kingpins are shown in Figure 8d. Figure 8e shows the estimated lateral distance between the vehicle and the lane line, and the estimated value tracks the reference value with little error. The road friction coefficient estimation results are shown in Figure 8f. If the reference value of the road friction is considered as 0.8, then the estimation accuracy was about 97.8%. Since the nonlinear estimator only works during steering, the estimation holds if the vehicle’s lateral acceleration is relatively small during DLC, for example, from 7 to 8 s. Compared with the slalom test results, the road friction estimation results dose not fluctuate because the lateral excitation is not continuous. From the experimental results, we can see that the road friction coefficient rapidly converges to the reference value.

## 5. Conclusions

In this paper, a nonlinear observer for the road friction coefficient during steering based on the self-aligning torque characteristics of the tires aided by vehicle lateral displacement information was proposed. A modified tire brush model was established according to the tire test data, and the model describes the tire characteristics more precisely than the original model. A nonlinear observer using vehicle lateral displacement information was designed, and the stability and robustness were analyzed. Experiments were conducted to verify the proposed road friction coefficient estimation algorithm. The test results demonstrate that the proposed method performs well during vehicle steering, and the estimated road friction coefficient converges to the reference value very rapidly. 

## 6. Future Work

We have modified the tire brush model according to the tire test data, and the results show that the modified model describes the tire characteristics properly. However, the tire tests were done with only one type tire in high friction condition, which is not sufficient to verify that the modified tire model is suitable for other tires with different sizes or types. Tire tests with more tires in different friction conditions should be conducted to verify the modified tire brush model.

The experiments were conducted only in high friction condition due to the test condition limitation. The algorithm should also be validated in low friction condition and high to low or low to high friction transition conditions in the future.

## Figures and Tables

**Table 1:** Vehicle parameters.

Parameters	Value
*m*/(kg)	1343.8
*b*/(m)	1.356
*l_f_*/(m)	1.112
*l_r_*/(m)	1.193
*I_z_*/(kg·m^2^)	1785
